# A computational fluid dynamics assessment of 3D printed ventilator splitters and restrictors for differential multi-patient ventilation

**DOI:** 10.1186/s41205-021-00129-1

**Published:** 2022-01-05

**Authors:** Daniel J. Duke, Alexander L. Clarke, Andrew L. Stephens, Lee Djumas, Shaun D. Gregory

**Affiliations:** 1 Department of Mechanical & Aerospace Engineering, Monash University, Clayton, 3800 Victoria Australia; 2 Department of Anaesthesia, Royal Women’s Hospital, Parkville, 3052 Victoria Australia; 3 Department of Anaesthesia and Pain Management, Royal Melbourne Hospital, Parkville, 3052 Victoria Australia; 4 CardioRespiratory Engineering and Technology Laboratory (CREATElab), Baker Heart and Diabetes Institute, Melbourne, 3004 Victoria Australia; 5 Department of Materials Engineering, Monash University, Clayton, 3800 Victoria Australia

**Keywords:** Ventilator splitting, Mechanical ventilation, Splitter, Restrictor, Airflow, COVID-19

## Abstract

**Background:**

The global pandemic of novel coronavirus (SARS-CoV-2) has led to global shortages of ventilators and accessories. One solution to this problem is to split ventilators between multiple patients, which poses the difficulty of treating two patients with dissimilar ventilation needs. A proposed solution to this problem is the use of 3D-printed flow splitters and restrictors. There is little data available on the reliability of such devices and how the use of different 3D printing methods might affect their performance.

**Methods:**

We performed flow resistance measurements on 30 different 3D-printed restrictor designs produced using a range of fused deposition modelling and stereolithography printers and materials, from consumer grade printers using polylactic acid filament to professional printers using surgical resin. We compared their performance to novel computational fluid dynamics models driven by empirical ventilator flow rate data. This indicates the ideal performance of a part that matches the computer model.

**Results:**

The 3D-printed restrictors varied considerably between printers and materials to a sufficient degree that would make them unsafe for clinical use without individual testing. This occurs because the interior surface of the restrictor is rough and has a reduced nominal average diameter when compared to the computer model. However, we have also shown that with careful calibration it is possible to tune the end-inspiratory (tidal) volume by titrating the inspiratory time on the ventilator.

**Conclusions:**

Computer simulations of differential multi patient ventilation indicate that the use of 3D-printed flow splitters is viable. However, *in situ* testing indicates that using 3D printers to produce flow restricting orifices is not recommended, as the flow resistance can deviate significantly from expected values depending on the type of printer used.

**Trial registration:**

Not applicable.

## Background

The SARS-CoV-2 pandemic and its associated disease COVID-19 has resulted in a surge of patients requiring mechanical ventilation in regions where demand for ventilators often exceeds supply. Advances in treatment for COVID-19 and improved understanding of the aerosol generation associated with alternative modes of oxygen administration have reduced the need for intubation compared to the first wave of the pandemic [1]. However, larger second and third waves of infections [2] have necessitated the intubation and mechanical ventilation of large numbers of patients, driving continued demand for ventilator equipment [3].

A possible solution to this problem is to split ventilators between multiple patients [4]. This approach was investigated in 2006 by Neyman et al. through the use of readily available Briggs T-connectors and human lung simulators [5]. The concept has since been investigated in several studies [6, 7].

Empirical evidence for the effectiveness of multi-patient ventilation is unclear [6]. Case reports of multi-patient ventilation exist where patients were exposed to the same parameters in volume [8] and pressure [9] control modes. Multi-patient ventilation is problematic and risky where patients have dissimilar ventilation needs [10] because each patient has different requirements for inspired oxygen concentration, airway pressure, inspiratory-expiratory ratio and respiratory rate. Cross-contamination between patients is also of concern [11].

In an unmatched pair of patients, differential multi-patient ventilation uses a flow restriction to reduce the inspiratory pressure to the patient with higher lung compliance [12]. Clarke et al. have recently demonstrated how this can be achieved by cutting and clamping an endotracheal tube to act as a simple flow restrictor [4]. However, the difficulty of balancing tidal volumes remains an issue [6], given the sensitivity of ad-hoc flow restricting devices and the limited availability of pressure and flow measurement sensors that can be inserted at the patient end of the circuit. In a disaster surge scenario, the availability of surplus T-connectors, hoses, tubing, connectors and transducers may also be problematic.

A solution to the aforementioned problems is the use of additively manufactured parts which are compatible with standard ventilator tubing and attachments. Various splitter designs have been proposed and have already been used in several countries [13].

For example, the authors have also developed a 3D printed flow restrictor which can be inserted in series with a 3D printed splitter to enable differential ventilation [14]. Fixed restrictors provide some potential advantages compared to variable valves/clamps. These include rapid production, low cost, local manufacture and predictable performance, which may remove the requirement for some difficult-to-source monitoring components [14].

A lack of empirical data to aid clinicians in selecting an appropriate flow resistor is a major barrier to practical implementation. Given the large number of possible designs and combinations of splitters and restrictors, the limited availability of suitable flow meters and pressure transducers in hospitals, and the limited time for implementation, *in situ* testing of every setup is not possible. To this end, researchers at the University of Bath developed a simple resistance-compliance (RC) model [15] which can be used to determine the required resistance in each circuit branch, including an online tool [16]. However, the use of these tools requires a priori knowledge of the resistance value an arbitrary 3D printed restrictor design will yield.

In this study, we have experimentally investigated the effect of 3D printer design and material on the resistance of a 3D-printed splitter and flow restrictor. We have considered a range of consumer and professional grade printers. This is motivated by the reported use of consumer 3D printers and non-medical grade materials during emergency situations in 2020, and ongoing charitable projects continuing this practice. We investigate whether consumer grade printers produce less accurate or repeatable pressure drop *in situ* when compared to professional grade printers.

In order to determine how the printed restrictor performance compares with expected performance, we have developed a *in silico* model of a 3D printed splitter and restrictor using open source computational fluid dynamics (CFD) software [17]. The model is capable of simulating a wide range of patients and restrictor sizes for 3D-printed splitters and restrictors of any design. The model indicates the expected performance of a ‘perfect’ restrictor and splitter whose internal geometry matches exactly with the stereolithography (STL) data used to generate the 3D printed parts.

## Methodology

### Component design and manufacture

**Fig. 1:**
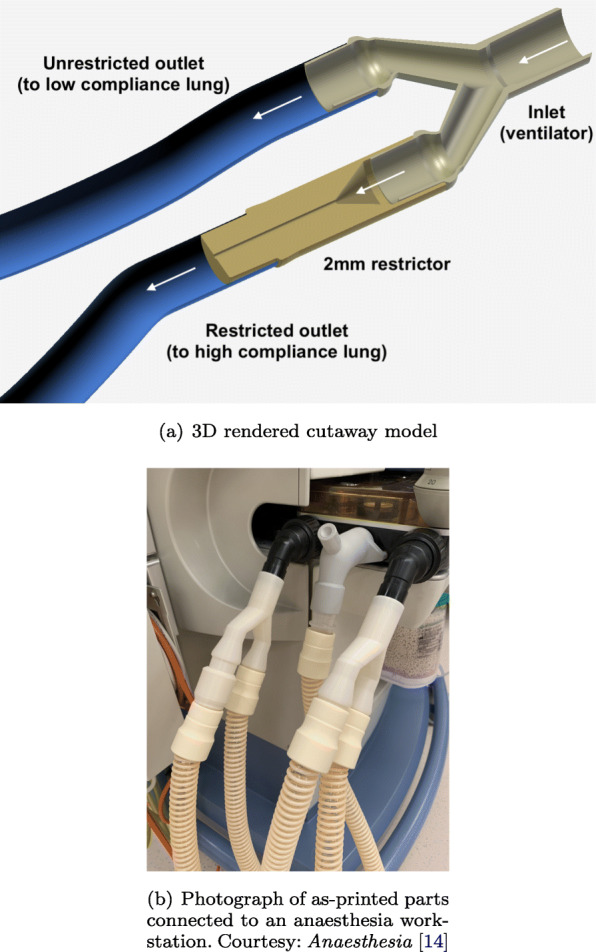
3D-printed splitter with restrictor on one port

The 3D-printed flow splitter and restrictor considered in this study uses standard 22 mm fittings, and can be inserted in series with a 3D-printed splitter to enable differential ventilation [14]. The 3D model (STL and original Fusion 360 design) files are freely available from the author’s website (https://alexanderclarke.id.au) and in the Supplementary Material. A computer rendering of the ideal geometry in a typical setup is shown in Fig. 1a. Figure 1b shows a photograph of a pair of splitters and restrictors connected to the inspiratory and expiratory circuits of an anaesthesia workstation.

**Fig. 2:**
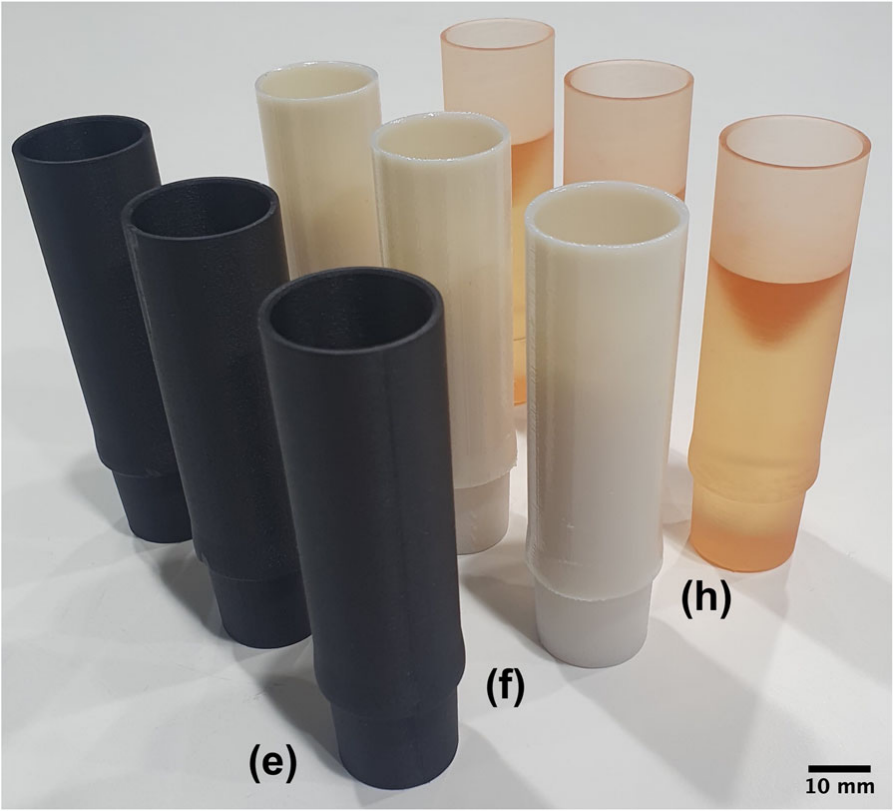
Samples of restrictors produced on professional grade 3D printers. Annotations match Figure 5

The 3D-printed parts were replicated using identical geometry data on 6 different professional and consumer grade printers with layer heights from 25 µm to 254 µm, as listed in Table 1. Two of the most common printing methodologies, fused deposition modelling (FDM) and photopolymer stereolithography (SLA) have been tested for both consumer and professional devices. Three different restrictor inner diameters (2, 3 and 4 mm) were considered for a total of 30 unique printed parts in the sample (several examples are shown in Fig. 2). These sizes span a typical range of desired pressure drops for practical applications. A completely filled experimental grid was used in the study, with all combinations of sizes and print materials considered with an equal number of replicates of each. Additional details regarding printer settings can be found in the Appendix.

**Table 1:** Summary of microscopy measurements for 3D printed ventilator restrictors with nominal internal diameter of *D*=2.00 mm, given in both absolute terms and as a ratio of the printer layer thickness

Manufacturer/Model	Type	Material	Layer thickness	True I.D.		Mean internal feature size	I.D. error to layer thickness ratio	
				Mean	Peak-peak variation		Mean	Peak-peak
(a) Creality LD-002R	Consumer SLA	Monocure 3D Rapid transparent photopolymer resin	50 µm	1.69 mm	0.06 mm	0.05 mm	2.4	1.0
(b) Prusa i3 Mk3S	Consumer FDM	PLA	50 µm	1.87 mm	0.04 mm	0.05 mm	3.0	0.8
(c) Prusa i3 Mk3S	Consumer FDM	PLA	100 µm	1.94 mm	0.12 mm	0.10 mm	1.2	1.2
(d) Prusa i3 Mk3S	Consumer FDM	PLA	200 µm	1.92 mm	0.15 mm	0.20 mm	0.8	0.8
(e) Stratasys uPrint	Professional FDM	ABS	254 µm	1.38 mm	0.14 mm	0.23 mm	2.7	0.6
(f) Stratasys Connex500	Professional Polyjet	Vero White Plus acrylate photopolymer	32 µm	1.74 mm	0.27 mm	0.43 mm	8.25	8.4
(g) Markforged Mark 2	Professional FDM	Nylon-carbon fibre composite	100 µm	1.78 mm	0.28 mm	0.10 mm	3.6	2.8
(h) Formlabs Form 2	Mid tier (Pro-sumer) SLA	Surgical guide resin	50 µm	1.73 mm	0.06 mm	0.05 mm	12.0	2.4

Microscopy measurement uncertainty is ±0.01 mm. Further details on printer settings may be found in the Appendix

### Experimental tests

The performance of the flow restrictors was measured *in situ* by connecting the upstream port to a dry compressed air supply. The steady state volumetric flow rate of air was measured using a NIST-calibrated pressure and temperature-corrected mass flow meter (M-50SLPM-D, Alicat Scientific, 0.6% accuracy). The pressure drop across the restrictor was measured using calibrated piezoelectric pressure transducers (PX119, Omega Engineering, 0.5% accuracy). Corrections for small variations in barometric pressure between simulations and experiments were made post-hoc. The flow rate was measured in the laminar flow region upstream of the restrictor. The upstream static pressure was measured from a static pressure tap located immediately upstream of the restrictor, and the downstream static pressure was measured at the end of a tube approximately 100 diameters in length in order to ensure that the flow was steady and fully developed. 200 repeated pressure and flow rate measurements were obtained for each restrictor.

At the conclusion of testing, samples were sectioned along the mid-plane using a linishing technique which slowly removes material to ensure that the sectioning process does not distort the internal geometry. They were inspected with a digital microscope at a uniform magnification of 7.5 µm / pixel to check for print errors, and identify surface features which may explain differences in their performance.

### Flow simulation

In order to evaluate the *in situ* performance of the splitter and restrictor, a control case was required. Since any experimental control will ultimately be biased by choice of material and manufacturing technique, a high fidelity computational fluid dynamics (CFD) model was used to provide the control. The simulation assumes perfectly smooth walls that conform exactly to the STL data used to produce the 3D printed parts.

Flow simulations were conducted using the *OpenFOAM* software package [18]. The software is open source, and freely available to researchers worldwide who may wish to reproduce the results shown here. Given the computational cost and complexity of the model, only the inspiratory circuit (left side of Fig. 1b) has been simulated.

We solved the governing equations for mass, momentum and energy of a compressible ideal gas flow [19] by time-marching the solution across a grid comprising 3.8×10^5^ hexahedral cells. This approach can be easily modified to simulate the flow through a 3D-printed part of any design or configuration.

The spatially resolved domain was limited to the 3D splitter and restrictor. The inlet conditions from the ventilator were simulated using empirical measurements obtained from a Hamilton C6S ventilator (Hamilton Medical, Bonaduz, Switzerland) in pressure control mode [4] with settings as shown in Table 2. The gas used in the simulation is a mixture of 80% O2 and 20% dry air. The gas composition and humidity do not manifestly affect the simulations or bench tests.

**Table 2:** Inlet and outlet conditions for the flow simulations: The three sets of compliance values show the range of values considered in this study

Ventilator (Inlet) conditions	
Peak ventilator pressure	30 cmH_2_O
Positive end-expiratory pressure	5 cmH_2_O
Respiratory rate (default)	16 breaths min^-1^
Pressure maximum time (default)	1.25 s
Target end-inspiratory volume	0.480 L per patient
Simulated gas properties	
Gas constant	R = 265 J kg^-1^ K^-1^
Gas specific heat capacity	936 J kg^-1^ K^-1^
Kinematic viscosity	2.02 10^-5^ Pa ·s
Patient breathing circuit (Outlet) conditions	
Unrestricted (stiff lung) compliance	C = 0.02 / 0.04 / 0.10 L ·cmH_2_O^-1^
Restricted (healthy lung) compliance	C = 0.04 / 0.08 / 0.20 L ·cmH_2_O^-1^
Patient lung and airway resistance	
*R* inspiration	13 cmH_2_O / (L ·s^-1^)
*R* expiration	12 cmH_2_O / (L ·s^-1^)
Endotracheal tube and breathing circuit properties	
*C*_*bc*_	0.004 L ·s cmH_2_O^-1^
*R*_*bc*_	22 cmH_2_O / (L s^-1^)

The simulations were parallelised on up to 150 CPUs using the MASSIVE computing facility at Monash University, Australia [20]. Each simulation typically requires 3500 CPU-hours to complete; equivalent to 3 days on a high-end desktop PC or less than 1 day on the cluster. We considered three restriction diameters (2.0, 3.0 and 4.0 mm), and a control case where there was no restrictor. We modelled a typical pressure maximum time of 1.25 s, as well as a longer inspiratory time to quantify the effect of inspiratory time extension on end-inspiratory volume. Further technical details regarding the simulations are provided in online supplementary material.

Rather than simulating the entire patient breathing circuit and airway in the finite volume solver, we use a lumped parameter model to simulate the response of a virtual breathing circuit to the inspiratory flow generated by the flow leaving each outlet port. Our model is similar to that of Plummer et al. [15]. Given than the inertial mass of the air inside the breathing circuit is negligible, the stagnation pressure *p*_0_ at the point where the breathing circuit connects to the splitter/restrictor outlet ports is related to the inspired volume *V*(*t*) by:

1 $$ p_{0}=\frac{\tau}{C} \frac{dV}{dt}+\frac{V}{C}.  $$

Where *C* is the compliance of the patient’s airway and lungs, and the time constant *τ* is:

2 $$ \tau=RC+R_{bc} \left(C_{bc}+C\right).  $$

*R* is the resistance of the patient’s airway, and *C*_*bc*_ and *R*_*b*_*c* are the compliance and resistance of the endotracheal tube and breathing circuit respectively. The breathing circuit stagnation pressure is related to the static pressure (i.e. the measured pressure) by Bernoulli’s equation:

3 $$ p_{0}=p+\frac{1}{2} \rho U^{2}  $$

where *U* is velocity magnitude and *ρ* air density. The flow in nearly all parts of the circuit can be considered to be incompressible, as the flow velocity is much smaller than the speed of sound. However, the flow inside the restrictor can reach relatively high velocities, necessitating consideration of air compressibility effects in order to correctly predict the pressure drop. This is achieved through the use of compressible mass conservation equations in the restrictor section [19] as opposed to the incompressible flow assumptions imposed by most lumped parameter models.

**Fig. 3:**
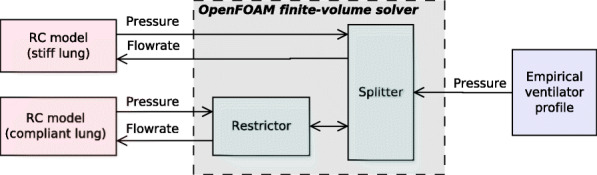
Schematic of the flow simulation showing the coupling of the inlet profile and the RC model for the patient’s breathing circuit to the finite-volume solver

Typical *R* and *C* values were obtained from literature [15, 21], as per Table 2. Three sets of patient airway and lung compliance values were modelled to investigate the sensitivity of the results to the compliance. Compliance can vary significantly between COVID-19 patients in practice, with deviations outside the typical range for ARDS [22]. The patient airway compliance was varied between the unrestricted and restricted ports of the splitter. The stiff lung model was connected to the unrestricted port (upper outlet in Fig. 1a) and the compliant lung model was connected to the restricted port (lower outlet in Fig. 1a). In this way, the RC model handles the patient and breathing circuit, and the finite-volume solver handles the splitter and restrictor, as shown in Fig. 3.

## Results

### Experiments

**Fig. 4:**
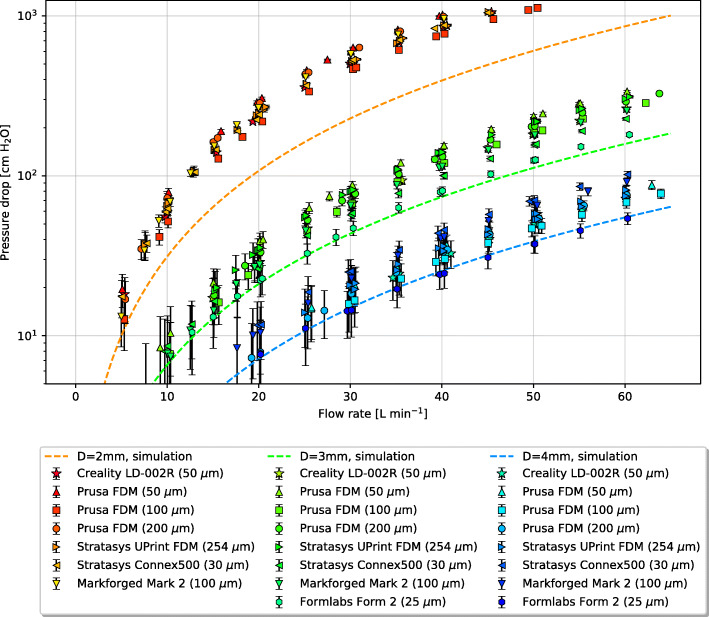
Raw experimental measurements (points) vs. simulation results (lines). The bracketed values indicate layer thickness, and error bars represent 95% confidence intervals around the mean pressure drop
- 33±10% of the deviation in pressure is estimated to be due to random errors in the wall geometry caused by the 3d print layers changing the wall roughness [19].
- the remaining small deviation is due to error in the simulations due to the choice of wall and turbulence models.

The experiments revealed that all the 3D printed parts had a consistently higher pressure drop than the simulated predictions (dashed lines). This result is shown in Fig. 4. The error was determined to be due to the following factors:

- 64±17% of the deviation in pressure is measured to be due to systematic error due to under-sizing of the internal diameter of the printed parts (see Table 1)

In all experiments, the measured flow rates and diameters at steady conditions correspond to pipe Reynolds Numbers in the range 5000 to 8000, where Reynolds Number is defined as

4 $$ \text{Re} = \frac{\overline{U} D}{\nu} \equiv \frac{4\dot{V}}{\pi D \nu}  $$

for pipe hydraulic diameter *D*, bulk velocity $\overline {U}$, volume flowrate $\dot {V}$ and gas viscosity *ν* (Table 2). This corresponds to the fully turbulent flow regime [19]. Roughness and imperfections in the nozzle wall will lower the transitional Reynolds Number in the restrictor further than in conventional smooth-wall pipes.

## Discussion

The challenge of 3D printing a flow restriction is that the pressure drop is extremely sensitive to the internal diameter. Dimensioning errors on the order of 10 µm can lead to significant changes in flow. For a simple flow restriction, the pressure drop may be expressed as a function of the volume flow rate $\dot {V}$, air density *ρ* and diameter *D* by:

5 $$ \Delta p=\frac{8\ \rho\ \dot{V}}{\pi^{2}{{c_{D}}^{2}\ D}^{4}}   $$

Where *c*_*D*_ is the variable discharge coefficient, which depends on the shape of the restriction and the Reynolds number (equation refReynolds). *c*_*D*_ has values typically in the range 0.6 to 1. If the restrictor diameter *D* changes slightly while $\dot {V}$ remains constant, such that *c*_*D*_ also remains constant, then the pressure drop will vary as the fourth power of the ratio of the true inner diameter to the nominal (design) diameter. This makes the pressure drop very sensitive to small changes in diameter.

Microscopy of the internal dimensions and features of the 3D-printed 2 mm restrictors, which suffer the largest relative diameter error, are given in Table 1. A complete table of results for all parts can be found in Table 6 in the Appendix. They reveal that the dominant cause of the high pressure drop is that the internal diameter is consistently under-sized relative to the nominal diameter. Undersizing of internal dimensions is a common problem experienced by all 3D printers due to the fact that they are generally calibrated to produce accurate external dimensions rather than internal dimensions. Any overspill of material (FDM) or UV light bleed (SLA) etc. will depend on the print settings (see Appendix) and the printer’s calibration algorithms. Since the majority of print jobs require external dimensional accuracy but can tolerate some error in the internal position of the perimeters and infills, internal surface errors can be many times larger than external surface errors. This effect has not been previously reported in studies of 3D printed ventilator components [13, 23] nor any other 3D printed medical devices developed during the course of the SARS-Cov-2 pandemic [24]. However, it has been observed in scientific research applications where 3D printed restrictors have been used to regulate fluid flow [25]. In previous reported applications where 3D printed holes were used to deliver cooling air to machinery, internal dimension tolerance errors were also found to be problematic [26].

FDM printers tend to undersize the internal features by 1–3 times the layer thickness due to extrusion of material into the void spaces. The severity of this effect depends on the extrusion speed and width, details of which can be found in the Appendix. If the road width is set by the printer so that the external face is correctly positioned as per the part file, the internal faces will be undersized.

Undersizing relative to the layer thickness is more pronounced in SLA printers. In addition to the aforementioned calibration bias, a layer of uncured resin is usually left inside the channel after printing. Most of this can be flushed out in post processing, but the rough surface allows a layer of resin to cling to the internal walls. Once cured, this decreases the internal diameter further.

Interestingly, the professional multi-material printer (Connex500) had the greatest degree of undersizing. This may be a result of the smooth acrylate material selected. While it provides a much smoother surface finish than the FDM parts, the benefits of this surface finish are outweighed by the tolerance on the internal dimensions. The use of polyjet printers to produce flow splitters of similar design has recently been published [27], but the undersizing problem has not been reported, likely due to the lack of investigation into the use of small flow restrictors.

**Fig. 5:**
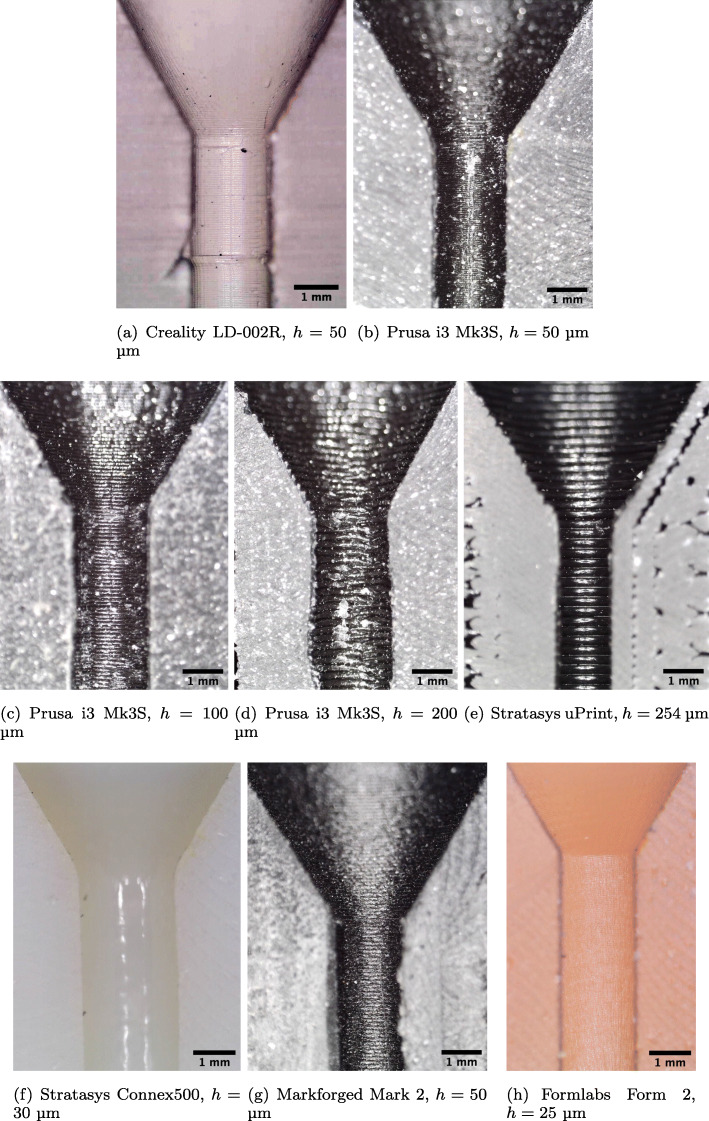
Sectioned microscope images of 2 mm flow restrictors, shown at a uniform magnification of 7.5 µm / pixel

The second cause of poor restrictor performance is the ridges inside the hole formed by the print layers. These are clearly visible in the FDM prints and consumer grade SLA prints in Fig. 5. The average size of the features was measured using Fourier analysis and the results are given in Table 1. Unlike a smooth wall, which allows the flow to form a parabolic profile, the ridges form a rough wall which drives the formation of a turbulent boundary layer. The low-velocity flow at the wall changes the displacement thickness of the boundary layer [19]. From a hydrodynamic perspective, this is equivalent to reducing the effective internal diameter of the restriction by several percent. Professional SLA and polymer jetting printers do not suffer from this problem to the same degree as consumer grade printers due to their smaller layer height.

A tertiary cause of variability in restrictor performance are imperfections in the print, which are particularly evident on consumer grade printers (Fig. 5a, d). These can lead to turbulent, unsteady flow which alters the friction and thus pressure drop.

### Flow simulations

Given the challenges associated with printing the parts, the question then arises as to whether these parts would perform acceptably if they could be manufactured to an acceptable dimensional tolerance. To answer this question, we consider the numerical flow simulations in more detail.

**Fig. 6:**
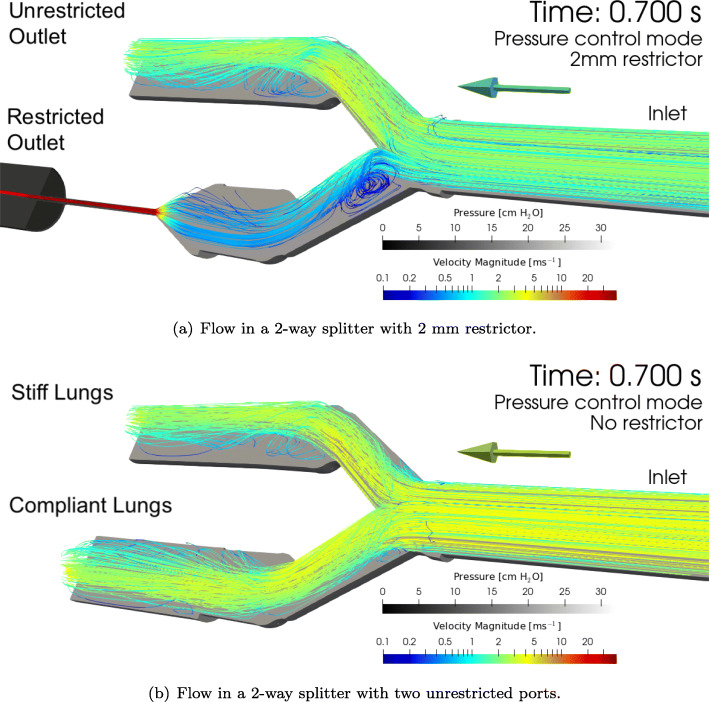
Visualisation of simulated flow at the peak pressure of the inspiratory cycle. The colour of the streamlines indicates the instantaneous magnitude of the local velocity, with the flow direction indicated by the arrow

Figure 6 show flow streamline visualisations from the simulations at the point of maximum pressure drop across the splitter and restrictor. The colour of the lines represents the local air velocity and the wall grayscale represents the local static pressure. With a 2 mm restrictor on one port (Fig. 6a), the flow is primarily directed into the unrestricted outlet, and a recirculation zone appears in the inlet to the restricted branch. With no restrictor, the flow is split more evenly between the two outlets (Fig. 6b).

**Fig. 7:**
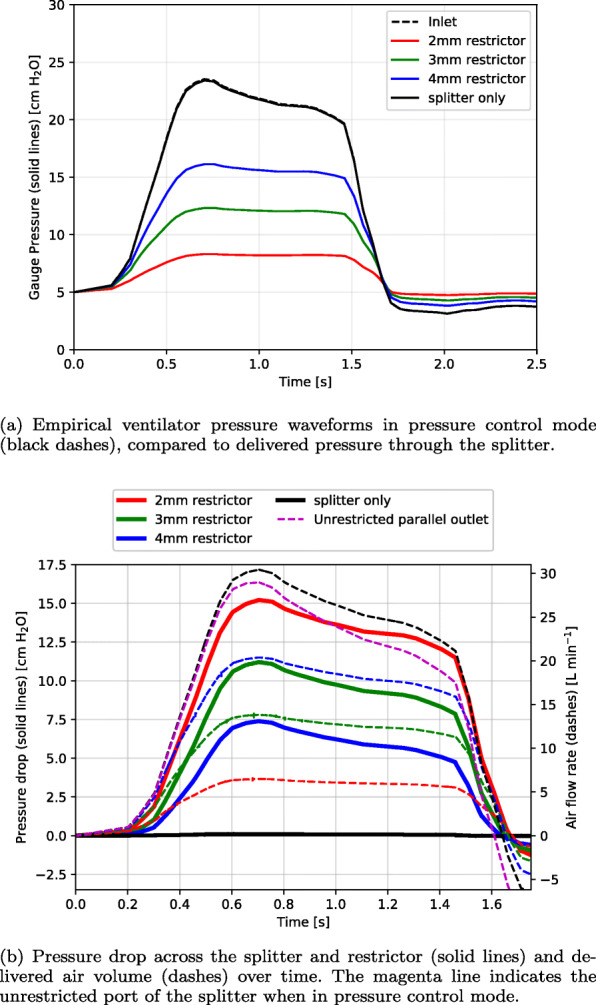
Simulated pressure waveforms over time with various restriction sizes, for (a) gauge pressure and (b) differential pressure drop

The pressures across the restrictor were obtained from the simulations by integrating over the inlet and outlet planes. The measured gauge pressures are shown in Fig. 7a, with the ventilator inlet pressure shown with a dashed line for comparison. The Y-splitter alone has a negligible effect on pressure loss given its large internal diameter.

Figure 7b shows the calculated pressure drop from the simulations (solid lines) with the volume flow rate indicated by the dashed lines. The magenta line indicates the flow rate for the second (unrestricted) branch of the splitter. It is relatively unaffected by the diameter of the splitter on the first branch. The black line indicates the behaviour of the restricted branch when the restrictor is removed. The difference between the two branches due to the differential patient airway compliances are apparent when comparing the black and magenta dashed lines.

Table 3 shows the pressure drop, flow rate and end tidal volumes corresponding to the results from Fig. 7a and b. The combination of splitter and restrictor pressure on the restricted outlet branch can be closely approximated as a polynomial function of the volume flow rate:

6 $$ \Delta p=a\dot{V}^{2}+b\dot{V}  $$

**Table 3:** Maximum pressure drop, flow rates and end-tidal volumes achieved using fixed 3D-printed flow restrictors in the inspiratory circuit

Restrictor diameter *D* [mm]	2.0	3.0	4.0	None
Peak pressure drop	15.22	11.21	7.40	0.11
(Simulated) [cmH_2_O]				
Peak flow rate	6.6	14.0	20.4	30.4
(Simulated) [L ·min^-1^]				
End tidal volume [mL]				
Healthy lung, restricted port				
C = 0.1 L ·cmH_2_O^-1^	123	245	362	503
Stiff lung, unrestricted port				
C = 0.2 L ·cmH_2_O^-1^	448	450	450	454

where the volume flow rate is L ·min^-1^, the pressure drop is in negative cmH_2_O, and the coefficients and correlation to the simulation data are given in Table 4. For restrictors of arbitrary diameter in the range *D*=2−4 mm, the pressure drop may be estimated with the following empirical formula:

7 $$ \Delta p \approx 3.55D^{-4.03}{\dot{V}}^{2}+10.27D^{-3.46}\dot{V}  $$

**Table 4:** Simulation resistance coefficients for two-way splitter and fixed restrictor for various restrictor diameters and lung-airway compliances

Restrictor diameter *D* [mm]	2.0	3.0	4.0	None
Pressure-flow coefficient *a*	0.22444	0.03961	0.01394	0
Pressure-flow coefficient *b*	0.88262	0.26081	0.07828	0.00361
Mean resistance [cmH_2_O/(Ls_-1_)]	156	51	25	0.22
Quality of fit [ *R*^2^]	0.99	0.99	0.99	0.90

*D* is given in mm, $\dot {V}$ in L ·min^-1^, and *Δp* in negative cmH_2_O. This formula fits all simulation results with *R*^2^=0.99 for flow rates up to 60 L ·min^-1^. It should not be used for restrictor diameters smaller than 2 mm or larger than 4 mm, and will not be accurate if the shape or design of the restrictor is modified. It does however account for systematic error in the printed component diameter, if the true diameter is measured (i.e. by plug gauge).

As the pressure drop is a quadratic function of the flow rate and the restrictor flow is compressible, the instantaneous resistance of the restrictor varies with flow rate and cannot be accurately represented by a constant. Equivalent mean resistance values at the time-average flow rate over the inspiratory cycle are given in Table 4 for reference purposes. A major benefit of the flow simulations presented here is the ability to handle nonlinear flow resistance and gas compressibility effects in restricted components where the velocities can be significant.

Additional simulations were carried out to assess the performance of the splitter-restrictor system across a range of airway compliances. While changes in compliance scale the end tidal volume, the pressure-flow coefficients depend only on the restrictor and splitter shape. This means that the end-expiratory volume can be controlled by simply adjusting the inspiratory period set on the ventilator. Further discussion of tidal volume control may be found in the Appendix.

### Limitations

Experimental testing and numerical simulation of 3D printed components is limited with respect to the variables which occur in clinical settings that cannot be easily incorporated into experimentation. These include, but are not limited to:

- Required variations in humidity and gas composition,
- Flow reversal or cross-flow due to spontaneous breathing effort by the patient,
- Leaks in fittings due to print tolerance errors on the external dimensions,
- Impact of the restrictor and splitter on the ventilator’s ability to alarm due to a fault in the patient circuit downstream of a small restrictor,
- Air leaks due to the porosity of some 3D printer materials, particularly FDM parts with low density infill,
- Degradation of 3D printing materials due to physical strain, absorption of moisture, and other environmental factors,
- Changes to recirculation caused by the addition of one-way valves and other components in the breathing circuit which have not been modeled in this study.

Further studies investigating the use of 3D printed components in clinical settings are recommended in order to ascertain the effect that these may have.

**Table 5:** 3D printer settings used in this study

Manufacturer / Model	Type	Material	Layer Thickness	Extrusion Temperature	Extrusion Width	Print Speed	Supports
(a) Creality LD-002R	Consumer SLA		50 µm	-	-	-	None
(b) Prusa i3 Mk3S	Consumer FDM	PLA	50 µm	215 ^∘^C	350–450 µm	30 mm/s	-
(c) Prusa i3 Mk3S	Consumer FDM	PLA	100 µm	215 ^∘^C	350–450 µm	30 mm/s	-
(d) Prusa i3 Mk3S	Consumer FDM	PLA	200 µm	215 ^∘^C	350–450 µm	30 mm/s	-
(e) Stratasys uPrint	Professional FDM	ABS	254 µm	310 ^∘^C	508 µm	Variable, typ. 60 mm/s	-
(f) Stratasys Connex500	Professional PolyJet	Vero White Plus acrylate photopolymer	32 µm	-	-	-	None
(g) Markforged Mark Two	Professional FDM	Nylon-Carbon Fibre Composite	100 µm	275 ^∘^C	400 µm	Variable, max. 280 mm/s	-
(h) Formlabs Form 2	Prosumer SLA	Surgical Guide Resin	50 µm	-	-	-	10 ^∘^angle, 1.0 mm density, 0.8 mm tip size

## Conclusion

This study aimed to assess the practical of 3D printed flow splitters and restrictors, to enable multi-patient support using a single ventilator. Deviations between the part design and the actual shape of the 3D printed parts due to the tolerance of the 3D printer and its software are the main barrier to practical implementation. The problem is particularly severe for small restrictors made on consumer-grade 3D printers. In practice, the risk of 3D printing tolerance leading to a change in flow resistance and pressure drop can be avoided:

1. through the use of an imposed inner diameter (i.e. fitting a piece of smooth-bore tubing into the throat of the restrictor or drilling out the hole to a known size after printing is completed),
2. by modifying the 3D printed part at the design stage to accommodate the known sizing errors produced by a particular 3D printer model through an experimental calibration such as the one described in this paper, or
3. placing a pressure transducer at the patient end of the circuit (where available) to measure the pressure drop and adjust the ventilator peak pressure accordingly to compensate for the increased resistance.

Using a novel computational fluid dynamics model, we were able to demonstrate that if the parts can be produced with the correct dimensions, they have the capacity to support multiple patients of varying lung capacity by adjusting the size of the restriction and the inspiratory time respectively. Restrictor diameters in the range of 4.0 mm down to 2.0 mm provide a practically useful range of pressure drops from 7.4 to 15.2 cm cmH_2_O under typical conditions.

The authors cannot recommend the use of 3D-printed flow restrictors or other flow-sensitive 3D printed devices without careful testing and *in situ* calibration, particularly where consumer or industrial printers are used in emergency scenarios. Ventilator splitting, whilst condoned by some jurisdictions (e.g. New York) during the COVID-19 pandemic comes with significant risk to both patients and staff. These risks must be balanced by the individual clinician, hospital and state in consultation with patients and their families.

This study demonstrates the complex fluid dynamics at work in these restrictors and highlights the limitations of a naïve implementation. At clinically relevant levels of flow restriction, these devices operate at the limits of the dimensional accuracy of even professional grade printers. The authors recommend extreme caution when utilising any type of ventilator-splitting apparatus, despite claims made by third-party commercial manufacturers of said devices regarding safety.

## Appendix

### Supplementary data

Table 5 details the printer settings used to produce the parts used in this study. Table 6 provides a complete summary of all the microscopy measurements beyond those highlighted in Table 1.

**Fig. 8:**
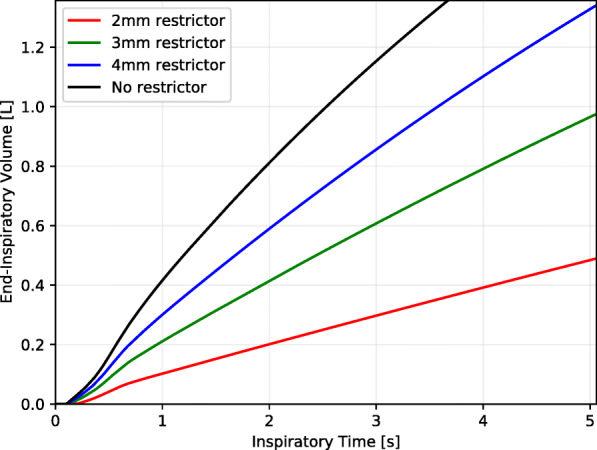
Titrating end-inspiratory (tidal) volume (vertical axis) by varying inspiratory time (horizontal axis) for several restrictor diameters, with airway and lung compliance of 0.1 L cmH_2_O^-1^

**Table 6:** Complete summary of all microscopy measurements made on restrictors 3D printed for consideration in this study

Manufacturer/Model	Type	Material	Layer thickness	Nominal I.D.	True I.D.		I.D. error to layer thickness ratio	
					Mean	Peak-peak variation	Mean	Peak-peak variation
(a) Creality LD-002R	Consumer SLA	Monocure 3D	50 µm	2.0 mm	1.69	0.06	2.4	1.0
		Rapid transparent		3.0 mm	2.60	0.13	8.0	2.6
		photopolymer resin		4.0 mm	3.48	0.17	10.4	3.4
(b) Prusa i3 Mk3S	Consumer FDM	PLA	50 µm	2.0 mm	1.87	0.04	3.0	0.8
				3.0 mm	2.86	0.05	2.85	1.04
				4.0 mm	3.90	0.13	2.05	2.56
(c) Prusa i3 Mk3S	Consumer FDM	PLA	100 µm	2.0 mm	1.94	0.12	1.2	1.20
				3.0 mm	2.95	0.06	0.55	0.55
				4.0 mm	3.99	0.16	0.15	1.55
(d) Prusa i3 Mk3S	Consumer FDM	PLA	200 µm	2.0 mm	1.92	0.15	0.8	0.80
				3.0 mm	2.84	0.03	0.81	0.16
				4.0 mm	3.86	0.03	0.69	0.16
(e) Stratasys uPrint	Professional FDM	ABS	254 µm	2.0 mm	1.38	0.14	2.8	0.60
				3.0 mm	2.81	0.03	0.73	0.10
				4.0 mm	3.84	0.09	0.63	0.35
(f) Stratasys Connex500	Professional PolyJet	Vero White Plus	32 µm	2.0 mm	1.74	0.27	8.3	8.4
		acrylate		3.0 mm	2.24	0.11	3.4	13.4
		photopolymer		4.0 mm	3.63	0.37	11.6	11.6
(g) Markforged Mark 2	Professional FDM	Nylon-cabon	100 µm	2.0 mm	1.78	0.28	3.6	2.8
		fibre		3.0 mm	2.91	0.10	0.87	0.95
		composite		4.0 mm	3.86	0.05	1.36	0.45
(h) Formlabs Form 2	Pro-sumer SLA	Surgical	50 µm	2.0 mm	1.73	0.06	12	2.4
		guide		3.0 mm	2.93	0.03	1.32	0.6
		resin		4.0 mm	3.91	0.05	1.77	0.94

Values are the average of multiple measurements made at the entry, mid-plane and exit of the restrictions for all replicate parts

### End-inspiratory (tidal) volume control

When using a fixed flow restrictor to generate differential inspiratory pressures, a means of independently titrating the tidal volume is required. This can be achieved through control of the inspiratory period at the ventilator. We repeated the simulation for increasing inspiratory times up to 5 seconds and cumulatively integrated the delivered volume over time to determine the end-inspiratory volume, including the leading and trailing transient effects produced by the splitter and restrictor. The resulting end-inspiratory volumes delivered through the restricted port of the splitter are shown in Fig. 8. As the restrictor diameter decreases, larger inspiratory times are naturally required to achieve an equivalent end-inspiratory volume.

## Data Availability

All supplementary material including open source computer software, multimedia and data from this study are freely available online at the authors’ website: http://github.com/djorlando24/ventilatorRestrictor
